# A Smart Shoe Insole to Monitor Frail Older Adults’ Walking Speed: Results of Two Evaluation Phases Completed in a Living Lab and Through a 12-Week Pilot Study

**DOI:** 10.2196/15641

**Published:** 2021-07-05

**Authors:** Antoine Piau, Zara Steinmeyer, Yoann Charlon, Laetitia Courbet, Vincent Rialle, Benoit Lepage, Eric Campo, Fati Nourhashemi

**Affiliations:** 1 Gerontopole University Hospital of Toulouse Toulouse France; 2 Laboratoire d'analyse et d'architecture des systèmes Centre national de la Recherche Scientifique Toulouse France; 3 Unité 1295 Institut National de la Santé Et de la Recherche Médicale Toulouse France; 4 Autonomie, Gérontologie, E-santé, Imagerie et Société Grenoble Alps University Grenoble France; 5 Department of Medical Information University Hospital of Toulouse Toulouse France

**Keywords:** frail older adults, walking speed, outpatient monitoring, activity tracker, shoe insert

## Abstract

**Background:**

Recent World Health Organization reports propose wearable devices to collect information on activity and walking speed as innovative health indicators. However, mainstream consumer-grade tracking devices and smartphone apps are often inaccurate and require long-term acceptability assessment.

**Objective:**

Our aim is to assess the user acceptability of an instrumented shoe insole in frail older adults. This device monitors participants’ walking speed and differentiates active walking from shuffling after step length calibration.

**Methods:**

A multiphase evaluation has been designed: 9 older adults were evaluated in a living lab for a day, 3 older adults were evaluated at home for a month, and a prospective randomized trial included 35 older adults at home for 3 months. A qualitative research design using face-to-face and phone semistructured interviews was performed. Our hypothesis was that this shoe insole was acceptable in monitoring long-term outdoor and indoor walking. The primary outcome was participants' acceptability, measured by a qualitative questionnaire and average time of insole wearing per day. The secondary outcome described physical frailty evolution in both groups.

**Results:**

Living lab results confirmed the importance of a multiphase design study with participant involvement. Participants proposed insole modifications. Overall acceptability had mixed results: low scores for reliability (2.1 out of 6) and high scores for usability (4.3 out of 6) outcomes. The calibration phase raised no particular concern. During the field test, a majority of participants (mean age 79 years) were very (10/16) or quite satisfied (3/16) with the insole's comfort at the end of the follow-up. Participant insole acceptability evolved as follows: 63% (12/19) at 1 month, 50% (9/18) at 2 months, and 75% (12/16) at 3 months. A total of 9 participants in the intervention group discontinued the intervention because of technical issues. All participants equipped for more than a week reported wearing the insole every day at 1 month, 83% (15/18) at 2 months, and 94% (15/16) at 3 months for 5.8, 6.3, and 5.1 hours per day, respectively. Insole data confirmed that participants effectively wore the insole without significant decline during follow-up for an average of 13.5 days per 4 months and 5.6 hours per day. For secondary end points, the change in frailty parameters or quality of life did not differ for those randomly assigned to the intervention group compared to usual care.

**Conclusions:**

Our study reports acceptability data on an instrumented insole in indoor and outdoor walking with remote monitoring in frail older adults under real-life conditions. To date, there is limited data in this population set. This thin instrumentation, including a flexible battery, was a technical challenge and seems to provide an acceptable solution over time that is valued by participants. However, users still raised certain acceptability issues. Given the growing interest in wearable health care devices, these results will be useful for future developments.

**Trial Registration:**

ClinicalTrials.gov NCT02316600; https://clinicaltrials.gov/ct2/show/NCT02316600

## Introduction

Frailty is an age-related syndrome characterized by a decline in biological reserves with an increased risk of impaired autonomy and death [1]. As such, implementing intervention programs promoting physical activity is essential in preventing functional decline [2-4]. To measure the efficacy of these programs, it seems necessary to monitor frailty indicators and adherence over time [5,6]. Frailty was defined according to the five Fried criteria (slow gait speed, low physical activity, unintentional weight loss, exhaustion, and muscle weakness) [1]. Participants with a score of 0 were robust, 1 to 2 were considered prefrail, and 3 to 5 frail.

Among these criteria, consistent data indicates that walking speed is one of the strongest to predict adverse outcomes [7,8]. Currently, a patient walking speed is evaluated during clinical consultations by manually measuring the time the patient takes to walk 15 feet. This discrete assessment often fails to detect changes in day-to-day walking speeds and does not reflect walking speeds in everyday environments. As such, continuous ambulatory monitoring would ensure precise monitoring of a patient’s health and better support medical diagnosis, especially by capturing a patient’s decrease in physical activity and walking speed profile [9].

Currently, physical activity assessments are often based on self-reported questionnaires with poor or inconsistent reliability [10,11], highlighting the importance of objective measures. The World Health Organization’s reports on aging and health propose the use of wearable devices to collect information on physical activity and gait speed as health indicators [5]. Digital technologies allow the monitoring of patient’s physiological data in their environment and thus tracking of subtle changes over time [9,12-14]. For example, accelerometers provide an objective measure of physical activity over a few days compared to standard physical performance measures [15]. Moreover, physical activity feedback with wearable sensors may also be incentive to increase daily activity [16-19].

Several sensor-based tools have been proposed to assess frailty and walking speed. However, they do not allow monitoring walking speed and activity in real-life conditions over long periods of time both indoors and outdoors [20,21]. To date, walking analysis research in patients who are frail is limited, and most studies involve electronic walkways, camera systems, or force plates, which limits real-life monitoring [9,22].

Thus, assessing a device specifically designed to monitor this population is relevant. Currently, multiple consumer-grade monitoring devices are commercialized, such as wrist-worn fitness trackers or smartphone apps. However, their accuracy is under debate especially in monitoring gait speed in older populations [19,23-25]. As such, their results must be interpreted cautiously. A majority of these devices evaluate activity by a built-in sensor counting one’s steps and thus does not help characterize a patient’s type of walk from normal walking to shuffling.

Our hypothesis is that a shoe insole is acceptable for long-term monitoring of indoors and outdoors walking speed in real-life conditions. A wireless insole is discrete and does not stigmatize the patient, users do not have to remember to wear it every day because it is placed in the patient’s walking shoes, and studies have shown that inertial feet sensors can accurately measure walking speed [25-27]. However, acceptability beyond a few hours of testing is not yet reported. The objectives of our multiphase study is to assess the technical feasibility (eg, wireless transmission and calibration protocol) and the acceptability from the user’s perspective. The secondary objective is to describe the evolution of a patient’s frailty syndrome and functional autonomy in both groups, quality of life, and health costs.

## Methods

### Study Design and Setting

In line with codevelopment and health technology assessment recommendations [28-30], we designed a two-phase pilot study [31] involving community-dwelling prefrail and frail older participants remotely followed at home. It consisted of, first, a noncomparative trial in a living lab and a 12-week prospective, parallel, randomized controlled clinical trial (field trial). The first trial lasted from September 2015 to January 2016. The second lasted from October 2016 to January 2019.

The living lab experiment was set up in a living lab at the University Institute of Technology in Blagnac, France (Maison Intelligente de Blagnac) located on the university campus [26]. This flat of 70 m^2^ is equipped with a networking infrastructure accessible to valid, frail, or disabled persons. It enables testing of technological devices in an environment similar to one’s home setting but with controlled technical conditions. The living lab phase was carried out in two subphases: (1) 9 older adults were evaluated in the Maison Intelligente de Blagnac living lab and (2) 3 older adults among the 9 participants of the living lab were followed up with at home for a month.

**Figure 1:**
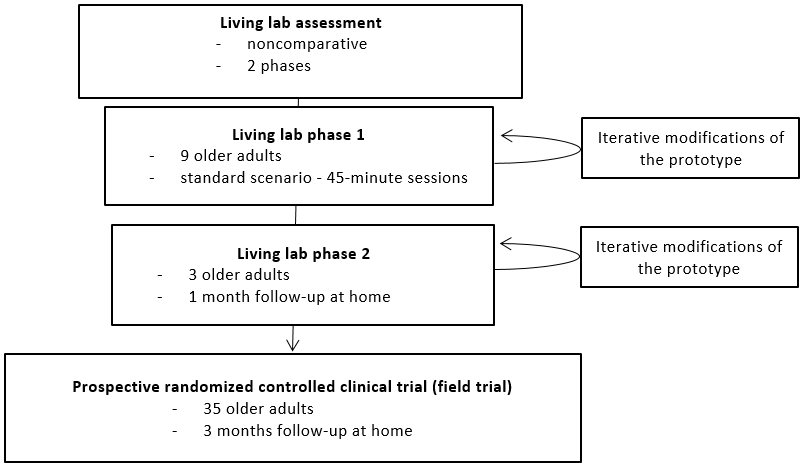
Description of the evaluation phases.

During the first phase, participants were invited to complete a single session standard scenario in the Maison Intelligente de Blagnac living lab (45-minute sessions consisting of a walking tour in the living lab and outside, as per usual) after calibrating the insole to their own step length and after a 10-minute presentation of the product’s objectives. During the second phase, volunteers were asked to use the device over a 1-month time period in their own homes (Figure 1). Participants were invited to wear the insole and use a touchpad without any additional instructions or training.

For the field trial, 35 participants were randomized following a 2:1 allocation into two distinct arms. Randomization was conducted independent of recruitment by the hospital’s epidemiology department. The aim of randomization was to describe the secondary objectives for future effect size calculation. Participants were randomized in either the intervention (smart insole follow-up) or control group (standard follow-up) and enrolled for 12 weeks.

### Intervention Description

**Figure 2:**
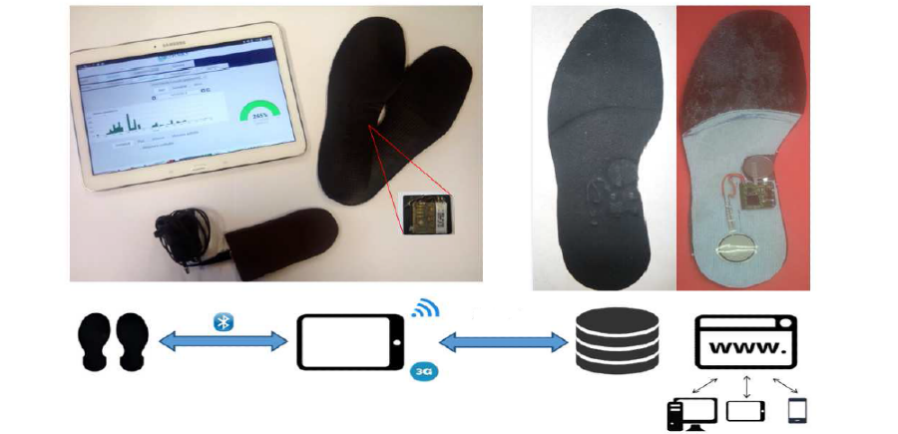
Overall technological device description. It includes a pair of insoles, an induction charger fitting in the shoe, a touchpad to collect data from the insole and provide feedback to the user, a secure remote database, and a web application for the patient and the physician. The insole is 2.5 mm at its thickest point (arch); it has a buffer memory and a flexible battery for walking comfort. If the battery is not recharged, an alert is issued to the user. The touchpad is presented here with a diagram of average walking speed and a diagram reporting active walking minutes.

**Figure 3:**
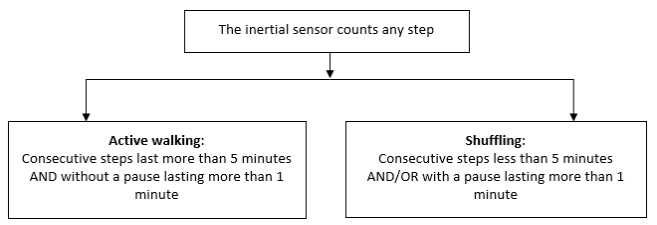
Active walking definition. The insole accounts for steps in any case.

Participants in the living lab phase and those randomized in the intervention group in the field trial phase were equipped with the instrumented shoe insole and were given a touchpad feedback app. The technological devices tested were a pair of insoles (only one insole is instrumented), a touch pad to collect data from the insole and inform the users about their activity (Bluetooth communication), and an induction charger to charge the shoe every night (Figure 2). The insole thickness is less than 2.5 millimeters at its thickest point (arch). The insole measures according to time (day, week, and month) the number of steps and walking distance, the average walking speed during walking periods, and active walking duration (as opposed to shuffling). Active walking was defined as continuous walking for at least 5 minutes with a tolerance of 1 minute (see Figure 3) considering that health benefits of aerobic exercise begin with any increase above the lowest levels of activity [32]. Moreover, the insole is calibrated on each participant’s step length for an accurate measurement of walking speed, unlike consumer-grade devices. The walking speed measure algorithms and the lab test results of the insole have been previously described [26].

Both groups benefitted from a frailty assessment at a geriatric day hospital for frailty (GHF) as per usual follow-up. Indeed, GHFs are developed nationwide in France following the French national authority recommendations and may accommodate up to five patients per day. Patients benefit from a follow-up phone call at 3 months and at 1 year by a nurse.

### Recruitment and Eligibility Criteria

Study participants were recruited through the Toulouse University Hospital GHF (France). Ethical approval for the study was obtained from the regional independent ethics committee in September 2014 (ID-RCB: 2014-A00523-440). The trial is registered on ClinicalTrials.gov (NCT02316600). All participants provided written consent. Baseline assessments were conducted in-person at the GHF. The inclusion criteria were (both phases):

- Patients 65 years or older living independently at home
- Activity of Daily Living (ADL) score (ranges from 0 to 6; the higher the score, the higher the level of functional autonomy in daily life is; eg, walking or dressing) [33] of 4 or higher
- Mini-Mental State Examination (MMSE; ranges from 0 to 30; the higher the score, the higher the level of global cognition) [34] of 24 or higher
- Prefrail or frail according to Fried criteria [1], for the field trial only

The sole exclusion criteria was life expectancy of less than 12 months. We did not request any specific level of computer literacy, and none of the participants were familiar with digital tools. There was no financial compensation for participation.

### Data Collection Procedure

At inclusion, sociodemographic data (gender, age, marital status, living place), frailty status [1], functional abilities with the ADL [33], and cognition with MMSE [34] were collected. For the field trial, other functional and physical scores were also assessed (Instrumental Activities of Daily Living [IADL] [35] and Short Physical Performance Battery [SPPB] scores [36], respectively).

In the living lab phase, semistructured interviews were conducted, focusing on technical feasibility and acceptability. We also scored the 3 *home participants’* satisfaction with the device at the end of the 1-month follow-up using the main themes identified during the interviews.

For the field trial, the primary outcome measured the acceptability of the device as follows:

- Qualitative indicators: acceptability assessment questionnaire based on the Quebec User Evaluation of Satisfaction With Assistive Technology scale [37] aimed to evaluate technology satisfaction degree from 1 to 5 (1: not satisfied at all; 5: very satisfied) and semistructured interview of the first 10 participants who completed follow-up.
- Quantitative indicators: number of days wearing the insole and average time of wearing the insole per day as declared by the participants and as measured by the insole and the number of connections to the app

The secondary outcome of the field trial measured:

- Frailty status according to the Fried criteria (slow gait speed, low physical activity, unintentional weight loss, exhaustion, and muscle weakness) [1]
- Lower extremity physical performance assessed with the SPPB (consisting of a balance test, a 3-meter gait test, and a 5 chair rises test; score ranging from 0 to 12; 12 indicating the highest degree of functioning) [36]
- ADL [33]
- IADL (ranging from 0 to 8; the higher the score is, the better the participant’s functional autonomy in daily life is; eg, driving) [35]
- Quality of life questionnaire (36-Item Short Form Health Survey [SF-36]; ranges between 0 and 100; greater score indicates better health-related quality of life) [38]
- EQ-5D-3L index, recording the patient’s self-rated health in a five-digit health state profile, each comprised of three levels (the score is converted into a single summary number ranging from −0.59 to 1; 1 indicating the highest level of perceived health) [39]
- Major medical events defined as any event leading to a hospitalization or an emergency admission
- Health costs

The semistructured interview was proposed to each participant at the end of the follow-up date (45 minutes for the living lab test, 1 month for the 3 living lab *home testers*, 3 months for the field test). The interview was face-to-face for the living lab group and by phone call for both home evaluations. Two researchers of the Age-Imaging-Modelization Laboratory (LC and VR; Joseph Fourier University Sociology Laboratory, Grenoble, France) conducted them. Each interview lasted 2 hours and was designed to explore key questions relating to acceptability. For the first phase of the study (living lab), nondirective exchanges were conducted to identify recurring themes. These themes guided the interviews of the field trial participants (n=10). All interviews were transcribed. The transcripts were analyzed using a conventional content analysis along with a summative qualitative content analysis [40].

For the field phase, a clinical research assistant also contacted participants at 1, 2, and 3 months to evaluate secondary outcomes. For acceptability, the following question was systematically asked: “how well did you tolerate wearing the insole during the past month?” (5 possible answers were proposed: from totally tolerable to totally intolerable).

### Statistical Analysis

For the field trial, we planned to include 35 participants complying with pilot study recommendations [41]. Qualitative variables were presented by effectives and percentages, quantitative variables by means and SDs. Likert-type items were handled as continuous variables.

Comparison tests were performed: quantitative variables with Wilcoxon-Mann-Whitney test and chi-square or Fisher tests for qualitative variables. Missing data was replaced with the mean values of the groups, allowing complete case analysis. A drawback of this approach is reduced variability and weakening of covariance and correlation estimates in the data.

The Department of Epidemiology of the Toulouse University Hospital in Toulouse conducted statistical analyses using Stata version 14.2 (Stata Corp).

## Results

### Living Lab

#### Study Sample Characteristics

A total of 9 participants were included, 6 (67%) women and 3 men. The mean age was 70.1 (SD 2.3) years (range 65-75), and none of them presented any functional disability (Table 1).

**Table 1:** Characteristics of participants (n=9).

Characteristics	Participants
Gender (female), n (%)	6 (67)
Age (years), mean (SD)	70.1 (2.3)
Activity of Daily Living score, mean (SD)	5.9 (0.3)
Mini-Mental State Evaluation score, mean (SD)	29.6 (0.5)
Frailty status (frail or prefrail), n (%)	1 (11)

#### Acceptability

The research assistant of the study informed participants in the living lab in the following terms: “Walking every day is beneficial to your health (e.g., independence, cognition) and the insole is designed to quantify your activity and provide feedback that can help you progress and make you want to walk more.” Interview feedback revealed participants’ understanding of the device’s objectives: 7 participants out of 9 cited *prevent the risk of becoming dependent* as a main objective, 5 *physical activity follow-up*, and 4 *motivate us to be physically active*. A total of 6 participants stated that wearing the insole could potentially encourage them to walk more. Indeed, real-time feedback would “motivate them to go out from home” and “stimulate their desire to adopt a healthier lifestyle.” A total of 4 participants suggested that reminder texts would increase physical activity motivation if activity fell below objectives. There were 5 other participants that were confident in achieving the walking threshold without any help. One person reported that such a reminder would be intrusive. None of the participants expressed concerned with the calibration protocol. Most participants (n=8) claimed that the insole was comfortable, light, and robust, and did not cause any discomfort. Nevertheless, flexibility and thickness were negatively highlighted by 2 of them with the fear of possible long-term use discomfort. Concerning the user touchpad interface, 5 participants expressed the need for a longer time practicing to assess acceptability. Several major themes were identified (example codes are given in parenthesis):

- Understanding and adhesion to the device’s objectives (eg, facilitating role)
- Device acceptability (eg, comfort)
- Device usability (eg, reliability)
- Device adherence (eg, time wearing the insole)

The 3 *home participants* reported wearing the insole for 1 month without early dropout. According to the insole data, they wore the insole for 22, 13, and 13 days out of 30, respectively. Total “active walking” time (as opposed to shuffling and therefore different from the number of steps) was 29 hours and 33 minutes (7288 average steps per day), 4 hours and 11 minutes (average steps per day 1748), and 3 hours and 52 minutes (average steps per day 1574), respectively. Average walking speed (calculated from the participants average step length obtained during the calibration protocol and cadence measurement) was 0.90 ms^–1^, 0.75 ms^–1^, and 0.69 ms^–1^.

All participants claimed that the insole was comfortable and that “once they were placed in the subject’s shoes, you tend to forget them.” According to users, the most interesting feedback information was walking distance (more than number of steps, walking speed, or the active walking time). Some texts of the interface were considered too small and colors not appropriate by 1 participant with age-related visual impairment. Of the 3 participants, 2 declared that they would use this device if it was commercialized. No harms or unintended side effects were reported. Table 2 summarizes the 3 *home participants*’ satisfaction with the device at the end of the 1-month follow-up. At the end of the interview, we asked them to rate their overall satisfaction with the device (score ranging from 0 “strongly disagree” to 6 “strongly agree”). The mean score was 3.1 (out of 6) for the *facilitating role*, 4.3 for *user friendliness*, and 2.1 for *reliability outcomes*.

Concerning technical feasibility, battery autonomy at home ranged from 30 to 48 hours, and no serious concern was raised about the induction charging system. Participants were satisfied with the daily routine, especially since there is no connection to be made for charging. One insole instrumentation was broken after 3 weeks of use. The 3 participants encountered synchronization problems between the insole and the touchpad (long latency).

**Table 2:** Participants satisfaction with the device at the end of the 1-month follow-up.

Participants satisfaction	Participant ratings^a^, mean (individual)
The device helps me to achieve my objectives (facilitating role)	3.7 (5, 3, 3)
It motivates me to complete my activities (facilitating role)	3.0 (1, 3, 5)
It helps me to be more efficient (facilitating role)	3.0 (5, 2, 2)
The device is easy to install (user friendliness)	2.7 (0, 5, 3)
The device is fun to use (user friendliness)	5.0 (5, 5, 5)
Using it is effortless (user friendliness)	5.3 (5, 5, 6)
I don’t need written instructions (user friendliness)	3.0 (1, 5, 3)
I easily learned to use it (user friendliness)	4.7 (5, 5, 4)
I quickly became an expert in its use (user friendliness)	4.7 (6, 4, 4)
It is easy to use (user friendliness)	4.0 (4, 4, 4)
It is user-friendly (user friendliness)	4.7 (5, 4, 5)
It is suitable for both frequent and infrequent users (user friendliness)	5.0 (5, 5, 5)
I always remember how to use it (user friendliness)	3.7 (6, 2, 3)
It is pleasant to use (user friendliness)	3.3 (5, 2, 3)
I am always able to use the device (reliability)	2.3 (1, 4, 2)
It always works as desired (reliability)	2.7 (3, 1, 4)
It always does exactly what I want (reliability)	1.3 (0, 2, 2)
It perfectly fits my needs	2.0 (1, 0, 5)
I need to have one	2.7 (1, 4, 3)
I will recommend it to a friend	3.0 (1, 3, 5)

^a^Answers range from 0 to 6: 0=strongly disagree, 1=disagree, 2=somewhat disagree, 3=no opinion, 4=somewhat agree, 5=agree, 6=strongly agree. The higher the rating, the better the satisfaction.

#### End Users’ Propositions

Participants proposed changing the insole’s design, the app, and the synchronization protocol. As a result, improvements were made before the field trial phase. To improve comfort and strength, the insole was modified by thermo-moulding, and the thickness of the electronics was reduced. The circuit was then varnished and encapsulated in epoxy glue and neoprene to protect it from impact and avoid friction. The participant’s interface was also modified to improve text and graphics readability. Upon user request, a light encoder was added on the induction charger that changes from red to green when the charger is rightly positioned in the shoe. Finally, the communication protocol between the tablet and the insole was optimized, and a bug disrupting data transmission (synchronization after several days of use) was fixed.

### Field Trial

#### Study Sample Characteristics

A total of 35 participants were included, 10 in the control group and 25 in the intervention group. In the intervention group, 6 participants left the study between visit one and visit two (3 because of defective equipment), 1 participant left the study between visit two and visit three because of defective equipment, and 2 participants left the study between visit three and visit four (1 for defective equipment).

**Figure 4:**
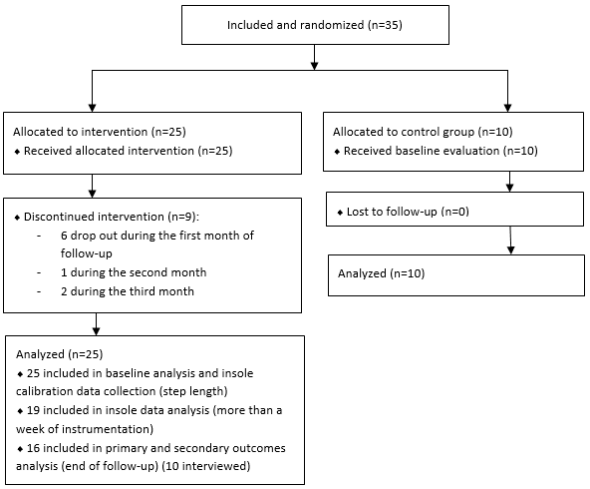
CONSORT (Consolidated Standards of Reporting Trials) flow diagram.

The CONSORT (Consolidated Standards of Reporting Trials) flow diagram is presented in Figure 4 [42].

A total of 80% (8/10) of participants were women in the *control* group and 64% (16/25) in the *intervention* group. The average age was 79 (SD 5.8, range 70-89) years. One-third lived in collective housing and two-thirds in individual housing. Participants were quite active; 72% (18/25) reported walking every day in the intervention group compared to 70% (7/10; *P*=.92) in the control one, and less than 10% reported not walking at all in both groups (Table 3).

**Table 3:** Characteristics of participants (n=35).

Characteristics		Control group (n=10)	Intervention group (n=25)	*P* value^a^	
Age (years), mean (SD)		77.8 (5.9)	79.3 (5.9)	.05	
Gender (female), n (%)		8 (80)	16 (64)	.44	
**Education, n (%)**					.44
	Low level	1 (10)	0 (0)		
	Middle level	5 (50)	12 (48)		
	High level	4 (40)	13 (52)		
**Marital status, n (%)**					.23
	Married	3 (30)	12 (48)		
	Single, divorced, widower	7 (70)	13 (52)		
**Living arrangements, n (%)**					>.99
	Alone (n=9 for control)	6 (67)	15 (60)		
	With other (n=9 for control)	3 (33)	10 (40)		
ADL^b^ score, mean (SD)		4.8 (1.5)	5.0 (1.4)	.79	
IADL^c^ score, mean (SD)		7.9 (0.3)	7.9 (0.2)	.59	
MMSE^d^ score, mean (SD)		27.0 (1.4)	29.1 (1.4)	.09	
SPPB^e^ score, mean (SD)		10.2 (2.8)	11.2 (1.1)	.42	

^a^Fisher test or Wilcoxon rank sum test.

^b^ADL: Activity of Daily Living.

^c^IADL: Instrumental Activities of Daily Living.

^d^MMSE: Mini-Mental State Evaluation.

^e^SPPB: Short Physical Performance Battery.

#### Acceptability Results (Primary Outcome)

##### Qualitative Indicators

Semistructured home interviews at the end of the 3-month follow-up (n=10) reported that the insole was well tolerated. A total of 10 (100%) participants declared wearing them every day. Most participants (n=7, 70%) affirmed that they did not need any incentive to wear the insole. Most claimed that the insole was comfortable, light, and robust, and did not cause any discomfort (n=7, 70%; 3 participants found them too thick). A total of 4 participants complained that the insoles did not fit in every type of shoe. The 3 participants who did not walk regularly (walk over short distances on a daily basis), declared that wearing the insole encouraged them to walk because “it stimulated their desire to surpass themselves” and “go out from home.” Concerning the user interface on the touchpad, users expressed some difficulties in handling the devices due to lack of habit (6 participants). As a result, they failed to read their data and did not understand its usefulness. These shortcomings led half of them (5/10) to almost abandon the tablet.

Regarding the tolerance of wearing the insoles during follow-up, 63% (12/19) of participants found it totally tolerable, 37% (7/19) quite tolerable at 1 month, 50% (9/18) and 44% (8/18) at 2 months, and finally increasing to 75% (12/16) and 19% (3/16) at 3 months. Concerning the acceptability questionnaire results at the end of the follow-up (n=16; Table 4), the overall answer was “quite satisfied” or “very satisfied.” The participants were “very satisfied” for weight; between “more or less satisfied” and “very satisfied” for dimensions, ease of adjusting, safety, robustness, and comfort; and less satisfied for ease of use and effectiveness in meeting their needs.

**Table 4:** Questionnaire results in the intervention group at the end of the follow-up (n=16).

How satisfied are you with (...) your device ?	Not at all, n (%)	Not very, n (%)	More or less, n (%)	Quite, n (%)	Very, n (%)
...the dimensions of...	0 (0)	0 (0)	3 (19)	4 (25)	9 (56)
...the weight of...	0 (0)	0 (0)	2 (12)	0 (0)	14 (88)
...the ease in adjusting...	0 (0)	0 (0)	2 (12)	4 (25)	10 (63)
...the safety of...	0 (0)	0 (0)	4 (25)	2 (12)	10 (63)
...the robustness...	1 (6)	0 (0)	3 (19)	3 (19)	9 (56)
...the ease of use...	1 (6)	4 (25)	1 (6)	4 (25)	6 (38)
...the comfort...	0 (0)	1 (6)	2 (12)	3 (19)	10 (63)
...the effectiveness...	4 (25)	1 (6)	4 (25)	1 (6)	6 (38)

##### Quantitative Indicators

A total of 25 participants were equipped with the insole, of which 6 for a duration of less than 7 days because of technical problems (Bluetooth communication with the touchpad) at the beginning of the study. Mean step length during calibration was 0.54 (SD 0.16) meters (n=25).

Apart from these 6 participants, 100% (n=19) of participants reported wearing the device every day at 1 month, 83% (15/18) at 2 months, and 94% (15/16) at 3 months of follow-up. Participants reported that the device was worn on average between 5.8 (SD 2.9), 6.3 (SD 6.4), and 5.1 (SD 3.7) hours per day at 1 month, 2 months, and 3 months, respectively. The mean number of days of wearing the insole according to the sensors data in a 3-month period was 29.2 (SD 28.7). If the participants with less than a week of instrumentation (n=6) were excluded, the mean increased to 40.4 (SD 28.8) without significant decline during follow-up (14.2, 12.7, and 13.5, respectively). On average, participants wore the insole for 5.6 (SD 3.7) hours a day. These figures only take into account the days when the insole effectively transmitted data to the server, which excludes connection failure periods or days with insufficient battery charging. The participants connected to the web application on average 45.4 (SD 68.3) times during the follow-up, which corresponds to a mean number of 4.3 (SD 10.6) connections per day.

#### Secondary Outcomes

##### Health Outcomes

For health outcomes, there were no statistically significant differences between the two groups at baseline and at the end of follow-up. At baseline, there were 0% (0/10) frail and 70% (7/10) prefrail in the control group, compared to 0% (0/10) and 83% (21/25), respectively, in the intervention group (*P*=.39). At the end of follow-up, 40% (4/10) frail and 40% (4/10) prefrail were found in the control group, compared to 19% (3/16) and 62% (10/16), respectively, in the intervention group (*P*=.58). Between visit one and visit four, 10% (1/10) improved their frailty status in the control group versus 19% (3/16) in the intervention group. However, these differences were not significant. Concerning the evolution of the physical activity criterion during follow-up, there was no significant differences despite a trend toward a more sedentary lifestyle in both groups. The overall EQ-5D score, SF-36 score, and functional scores (ADL, IADL, and SPPB) did not show any significant difference between the two groups.

Two notable adverse events not attributable to the intervention were reported, one in each group: 1 participant in the control group had a fall and 1 participant in the intervention group had a fracture.

##### Health Costs, Installation, and Maintenance Costs

Intend to treat analysis results showed a trend in favor of the intervention group in terms of costs (Table 5).

**Table 5:** Descriptive cost data.

Cost data	Control	Intervention	*P* value
Medical visits total costs (€^a^)	2001.00	1051.00	N/A
Hospitalization total cost (€)	15,374.60	6751.10	N/A
Total costs (€)	17,375.60	7802.10	N/A
Medical visits, n	87	45	N/A
Hospitalization stays, n	11	7	N/A
Medical visits cost (€), mean (SD)	111.20 (124.8)	27.70 (120.9)	.03
Hospitalization cost (€), mean (SD)	854.10 (1245.20)	177.70 (1202.80)	.21
Total cost (€), mean (SD)	965.30 (1329.90)	205,32 (1284.90)	.049

^a^A currency exchange rate of €1=US $1.2 is applicable.

A commercial company (SADIR Assistance) set up home installation and calibration protocol. They spent on average 33.9 (SD 13.1) minutes per visit per person during the study, for an average number of 3.8 (SD 0.6) visits per person (including the installation visit). The mean cost for installation and maintenance communicated by the company was €47.7 (SD €9.7) per month per person (a currency exchange rate of €1=US $1.2 is applicable).

## Discussion

### Principal Results and Limitations

Our 12-week field trial is the first to assess participant’s acceptability of an instrumented insole over 12 weeks in frail older adults in a real-life setting. Difficulty of physical activity follow-up in older individuals and insufficient in-person measures and self-reported data limit clinical research in this domain [9,22]. Currently, there is limited data on the use of instrumented insoles beyond a few hours of laboratory testing.

This study confirmed the importance of a multiphase design for health technologies. The living lab participants valued the study’s participative aspect and proposed modifications concerning both the software and the hardware, including the manufacturing method of the insole. Our living lab tests introduced an *optimized reality* between technical lab tests [26] and an ongoing *real-life* field trial. User feedback provided technical and acceptability issues, which were fixed before the field trial (low reliability scores, mainly associated with synchronization problems between the insole and the touchpad). However, despite these precautions, certain technical problems affected the field trial.

Participants validated the device’s design: induction charger, charging routine, battery autonomy, and data transfer automation. The insole calibration phase raised no user concern. These are important results as the device was specifically designed to ensure its unobtrusiveness. There were several reasons to choose an insole: it is unobtrusive and can be worn without disturbing or stigmatizing the person, several studies showed that inertial sensors worn on the feet allow accurate walking speed measurement [27,43,44], and the user does not need to remember wearing it.

Semistructured interviews and questionnaires at the end of follow-up reported that the insole was actually worn, unobtrusive, and well tolerated. Moreover, participants were compliant during the 3-month follow-up; this was confirmed by objective data measured by the insole. Most participants claimed that the insole was comfortable and did not cause any discomfort. This result was innovative, as it is one of the first to describe insole long-term wearing acceptability in older adults who are frail. Thus, a thin instrumentation including a flexible battery was designed. This technical challenge remains and must be considered when instrumenting an insole because it is one of the main participant complaints. Acceptability of wearable devices is the cornerstone of large implementation in real-life settings. Finally, those who did not walk regularly also expressed the fact that wearing a smart insole could encourage walking, which remains to be proven.

We found no significant differences between groups in terms of physical activity or frailty evolution. This could be explained by the low study power and by the fact that our touchpad did not offer any incentive or educational content. Information and communication technology–supported lifestyle programs and motion sensing–based monitoring can influence daily physical activity thanks to feedback [45]. However, adding educational content is more efficient [46,47].

This study included a small sample of volunteer older participants. Even if a few exclusion criteria were applied, there was probably a recruitment bias due to highly motivated participants. Moreover, a large proportion of participants in the intervention group discontinued the intervention because of technical issues.

The results obtained by this semiqualitative approach are not free of the usual biases, in particular external validity. There are other limitations to the generalizability of our results. Certain participants brought up the difficulty of integrating an insole in their shoes (eg, sandals), which could be even more problematic in countries where people walk barefoot indoor or wear outdoor shoes.

Moreover, the use of the touchpad interface was not satisfying because of lack of technical support for using it and participant’s computer literacy, web interface ergonomic issues, and the absence of educational content (eg, video tutorial). These shortcomings led most of the participants not to use the touchpad. The motivational aspect of our device was poor despite the potential interest this could have [48]. In a second development phase, an educational and motivational network should be developed to increase user’s adherence. It seems important to also include participant’s computer literacy when developing health devices in this population to ensure better acceptability.

Previous work has highlighted the difficulty of implementing such technical devices in health care practice due to the importance of material and human investment [49]. The results of this study give a glimpse into health device development and improvement, but further studies are required in a larger population to improve large-scale implementation.

### Comparison With Prior Work

Previous studies have shown the possibility to monitor mobility-related activities based on motion sensors, but few explicitly mentioned acceptability issues or used experimental research designs to evaluate clinical applications in older people [16,22,50,51]. Most systems consisting of multiple sensors or devices are difficult when applied in long-term monitoring in real-life (eg, the DynaPort MoveMonitor weighs 44.5 g and is fixed with an elastic belt [15]). A few studies evaluated accelerometer-based devices on short treadmill walks [52] on a short time period at home, ranging from a few minutes to days [15,53-56] with up to 20 older participants with unknown frailty status.

Most of these studies explore various aspects of walking such as depth posture and activity detection. Nevertheless, none of these studies evaluated long-term acceptability of such wearable devices in monitoring walking activity in an older participant sample.

Kaye and colleagues [57] conducted the most notable study. They evaluated in-home walking monitoring using embedded sensors in autonomous living older individuals during a 4-week period. The study did not specifically target frailty status monitoring and was limited to indoor activity. They were able to accurately monitor mean walking speed and even predict falls [9]. Embedded and wearable tools to monitor walking activity provide complementary information. Outdoor walking speed analysis has advantages on indoor measurements because walking distance may be longer and informs global aerobic physical activity [22,58].

Consumer-grade activity trackers (connected devices or smartphone apps) are increasingly popular. However, there is a lack of real-life research on the device’s performance in older adults. Given the inaccuracy of these applications, caution is required in promoting self-monitoring physical activity and their use for health prediction [19,23,24]. Indeed, the absence of step length calibration does not allow accurate measures of walking speed and walking characterization. Another major concern is their lack of acceptability in frail older adults. Most of them require minimal computer literacy, and users have to remember to wear it every day. Moreover, there is many constraints related to obtrusiveness (eg, device charging and data transfer). Lastly, the algorithms used to measure steps and other metrics are typically proprietary and may not be available to investigators [59].

### Conclusion and Perspectives

Wearable connected sensors are promising for real-life monitoring and appear to be a solution in improving physical activity promotion in frail older adults. However, optimal deployment of wearable health devices will require further research conducted in real-life conditions to test acceptability, effectiveness, and costs. This study reports real-life acceptability data on an instrumented insole in frail older participants over a 12-week period. These results are informative in terms of technical choices for those who wish to instrument a shoe insole.

This field of research is essential and offers interesting perspectives. Along physical activity promotion, these tools would improve detection of early preclinical health transitions implicated in decreased physical performance [9,57,60,61] (eg, gait speed variability over time). Thus, continuous measurements would also enable identification of innovative “digital biomarkers” as a complementary solution to “traditional” biomarkers, leading to more personalized interventions. 

## Abbreviations

ADL: Activity of Daily Living
CONSORT: Consolidated Standards of Reporting Trials
GHF: geriatric day hospital for frailty
IADL: Instrumental Activities of Daily Living
MMSE: Mini-Mental State Examination
SF-36: 36-Item Short Form Health Survey
SPPB: Short Physical Performance Battery
